# Arteriovenous Malformation in Temporal Lobe Presenting as Contralateral Ocular Symptoms Mimicking Carotid-Cavernous Fistula

**DOI:** 10.1155/2013/158961

**Published:** 2013-02-21

**Authors:** Fadzillah Mohd-Tahir, Ishak Siti-Raihan, W. H. Wan Hazabbah

**Affiliations:** Department of Ophthalmology, School of Medical Sciences, Universiti Sains Malaysia, 16150 Kubang Kerian, Kelantan, Malaysia

## Abstract

*Aim*. To report a rare case of arteriovenous malformation in temporal lobe presenting as contralateral orbital symptoms mimicking carotid-cavernous fistula. *Method*. Interventional case report. *Results*. A 31-year-old Malay gentleman presented with 2-month history of painful progressive exophthalmos of his left eye associated with recurrent headache, diplopia, and reduced vision. Ocular examination revealed congestive nonpulsating 7 mm exophthalmos of the left eye with no restriction of movements in all direction. There was diplopia in left lateral gaze. Left IOP was elevated at 29 mmHg. Left eye retinal vessels were slightly dilated and tortuous. CT scan was performed and showed right temporal arteriovenous malformation with a nidus of 3.8 cm × 2.5 cm with right middle cerebral artery as feeding artery. There was dilated left superior ophthalmic vein of 0.9 mm in diameter with enlarged left cavernous sinus. MRA and carotid angiogram confirmed right temporal arteriovenous malformation with no carotid-cavernous fistula. Most of the intracranial drainage was via left cavernous sinus. His signs and symptoms dramatically improved following successful embolisation, completely resolved after one year. *Conclusion*. Intracranial arteriovenous malformation is rarely presented with primary ocular presentation. Early intervention would salvage the eyes and prevent patients from more disaster morbidity or fatality commonly due to intracranial haemorrhage.

## 1. Introduction

Intracranial arteriovenous malformations (AVMs) are cerebrovascular lesions which consist of networks of arterial and venous channels which communicate directly without any intervening capillary bed. These abnormal communications are divided into two types, plexiform and fistulous. In plexiform type, one or more arterial channels feed a core of tightly venously loop or a nidus, while in the fistulous type, an arterial channel empties directly into a venous channel or the lesion is diffuse with anomalous vessels dispersed among normal brain parenchyma without a nidus. The fistulous types are also known as dural AVMs, which are supplied by meningeal branches of external carotid artery. In contrast, the plexiform type is supplied by branches of the cerebral or cerebellar arteries and therefore also known as pial AVMs. Acquired carotid-cavernous sinus fistula is the most common types of AVM encountered by Ophthalmologist [1]. Patients presented with ocular symptoms resulted from abnormal communication between arterial and venous channels within the cavernous sinus. Ocular symptoms rarely become primary manifestation of intracranial AVMs [2].

## 2. Case Report

A 31-year-old Malay gentleman presented to our Ophthalmology clinic with history of progressive bulging of the left eye associated with redness and pain for 2 months. He has no known medical illness although his blood pressure was reported slightly abnormal on his last visit to general practitioner. For the past five years, he has been having recurrent nasal blocked which resolved by nasal decongestion spray that he bought over the counter. He started to have recurrent headache since his last episodes of nasal congestion which was three months prior to his visit to us. In the past one month, he has reduced vision in the left eye and noticed double vision on turning to the left.

**Figure 1:**
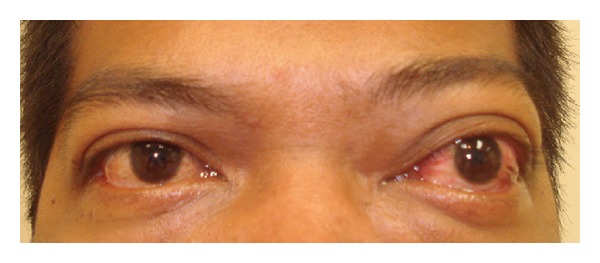
Congestive nonpulsating exophthalmos of the left eye.

**Figure 2:**
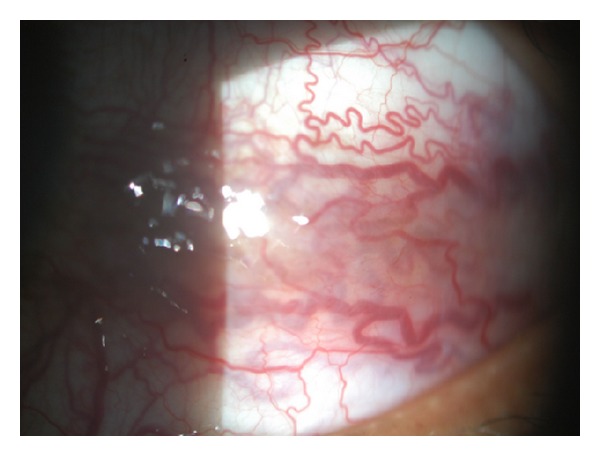
Dilated and tortuous conjunctival vessels.

His visual acuity in the left eye was 6/18 and 6/9 in the right eye. Ocular examination revealed congestive nonpulsating 7 mm exophthalmos of the left eye, measured by Hertel exophthalmometer (Figure 1). There was no restriction of eye movements in all direction of gazes but there was diplopia in left lateral gaze. There was no restriction of eye movements in all direction of gazes. At primary gaze intraocular pressure was 21 mmHg in the right and 29 mmHg in left eye, with no difference in other gaze. Fundoscopy of the left eye showed slightly dilated and tortuous retinal vessels (Figure 2). The right fundus was normal. There was no optic disc swelling on either eye. OCT pupillometry was normal. Colour vision and visual field on each eye were normal. Systemic examinations were normal except a borderline blood pressure. There was no sign of thyroid disease. Cranial nerves were intact. He is moderately overweight but there was no sign of increased intracranial pressure.

**Figure 3:**
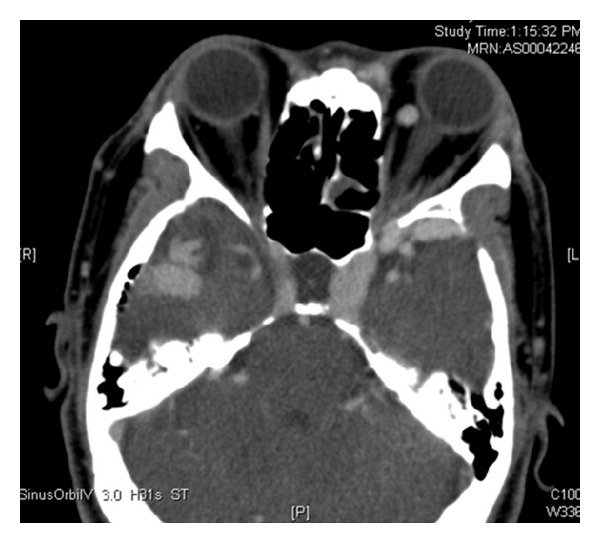
Enlarged cavernous sinuses and dilated left superior ophthalmic vein.

**Figure 4:**
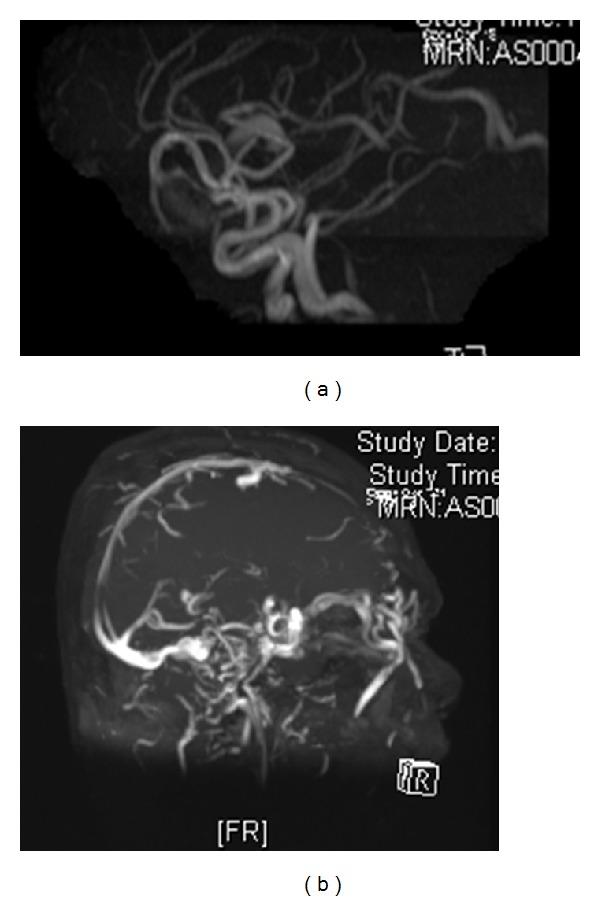
MRA confirmed right temporal arteriovenous malformation with no carotid-cavernous fistula.

CT scan was performed and showed right temporal arteriovenous malformation with right middle cerebral artery as feeding artery. The left superior ophthalmic vein was tortuous and dilated measuring 0.9 mm in diameter. The left cavernous sinus was enlarged (Figure 3). Extraocular muscles of left eye were relatively larger than the right eye. MRA and carotid four vessels angiogram confirmed right temporal arteriovenous malformation with a nidus of 3.8 cm × 2.5 cm (Figure 4). Arterial feeder is middle cerebral artery with no supply from external carotid artery. There was no aneurysm and no carotid-cavernous fistula. The right temporal AVM is grade 4 based on Spetzler Martin grading system. There was severe venous hypertension with most of the intracranial drainage being via left cavernous sinus. Venous drainage of the AVM is via the dilated and tortuous right temporal cortical vein into the right transverse sinus. There was a tight stenosis at the right sigmoid sinus. Hence from right transverse sinus, there was retrograde flow into superior sagital sinus and straight sinus that reach the left cavernous sinus and finally into the left superior orbital vein and left petrosal sinus. Left sigmoid sinus is occluded. 

His proptosis dramatically improved following successful embolisation of the arteriovenous malformation. Follow-up after one year showed that the uncorrected visual acuity of 6/9 improved to 6/6 after refraction. Following cessation of antiglaucoma eye drops, his IOP remains between 14 mmHg and 16 mmHg on several visits. The proptosis has completely resolved.

## 3. Discussion

Plexiform AVMs, which is more common than dural AVM, are almost exclusively congenital and tend to be asymptomatic until later in life. Half of the cases presented as intracranial haemorrhage with mean age at diagnosis being 31 years old [3]. About quarter of all intracranial AVMs come into medical attention as seizures while the remainder manifest as chronic headache or focal neurological deficits. Both plexiform and dural types of intracranial AVM are rarely presented with primary ocular symptoms because orbital drainage from cerebral AVM is rare. 

In a study over 4-year duration done by Volpe et al., only 3 out of 100 adult patients with cerebral AVM have orbital drainage as their main venous drainage [4]. The ocular manifestation varies, depending on the location of the AVM. One of the patients in the study had AVM at unilateral temporal-parietal lobe while another patient had AVM at unilateral parietal-occipital lobe region. Both patients had bilateral optic nerve involvement although asymmetry and worse on the ipsilateral side of the AVM. The third patient had AVM at parietal lobe and presented with headache only with no visual symptoms and had a normal neuro-ophthalmic examination. To our knowledge, this is the first report of plexiform (pial) AVM presented with contralateral globe proptosis and vascular engorgement as a result of rerouting of venous outflow through contralateral orbital venous drainage. Fortunately, our case had no optic nerve swelling or atrophy and the intraocular pressure back to normal range following the embolisation of his AVM.

In plexiform AVMs, venous stenosis or occlusion is believed to be the consequence of chronic endothelial changes due to turbulent and irregular flow [5]. In our case, the tight stenosis at the right sigmoid sinus caused the venous flow retrogradely to the anterior part of the brain, while the total occlusion of left sigmoid sinus caused severe venous hypertension; hence most of the intracranial drainage was via left cavernous sinus. Perhaps the abnormal rerouting of the venous flow to the contralateral side rather than to the ipsilateral orbital drainage had distributed the pressure and delayed the optic nerve involvement in this case. Successful embolisation of the AVM has reduced the intracranial venous hypertension and subsequently reduced the intraocular pressure.

## 4. Conclusion

Ophthalmologist may be the first physician to encounter clinical manifestations of intracranial arteriovascular malformation that may herald devastating neurologic complications. Although rare, prompt diagnosis facilitates early and most appropriate management and therapy, early intervention would not only salvage the eyes but also prevents patient from more disaster morbidity or fatality commonly due to intracranial haemorrhage.
